# Limited predictive value of blastomere angle of division in trophectoderm and inner cell mass specification

**DOI:** 10.1242/dev.103267

**Published:** 2014-06

**Authors:** Tomoko Watanabe, John S. Biggins, Neeta Bala Tannan, Shankar Srinivas

**Affiliations:** 1 Department of Physiology Anatomy and Genetics, University of Oxford, Oxford OX1 3QX, UK; 2 Cavendish Laboratory, University of Cambridge, Cambridge CB3 0HE, UK

**Keywords:** Inner cell mass, Trophectoderm, Mouse embryogenesis, Pre-implantation lineage specification, Time-lapse microscopy

## Abstract

The formation of trophectoderm (TE) and pluripotent inner cell mass (ICM) is one of the earliest events during mammalian embryogenesis. It is believed that the orientation of division of polarised blastomeres in the 8- and 16-cell stage embryo determines the fate of daughter cells, based on how asymmetrically distributed lineage determinants are segregated. To investigate the relationship between angle of division and subsequent fate in unperturbed embryos, we constructed cellular resolution digital representations of the development of mouse embryos from the morula to early blastocyst stage, based on 4D confocal image volumes. We find that at the 16-cell stage, very few inside cells are initially produced as a result of cell division, but that the number increases due to cell movement. Contrary to expectations, outside cells at the 16-cell stage represent a heterogeneous population, with some fated to contributing exclusively to the TE and others capable of contributing to both the TE and ICM. Our data support the view that factors other than the angle of division, such as the position of a blastomere, play a major role in the specification of TE and ICM.

## INTRODUCTION

The mouse zygote undergoes near synchronous cleavage divisions to give rise to a morula composed of eight roughly spherical blastomeres. These cells become polarised along their radial axis with distinct apical (surface) and basolateral (central) domains. One of the clearest manifestations of this is compaction, whereby the surface of the morula becomes smoother because blastomeres adhere more closely to one another. All blastomeres of the compacted eight-cell morula are equivalent, having an apical face exposed to the outside environment and a basolateral face in contact with surrounding blastomeres. Upon further division, two distinct populations of cells arise – polar ‘outside’ cells with an apical face exposed to the outside and apolar ‘inside’ cells, surrounded by outside cells and therefore embedded completely within the embryo. The trophectoderm (TE) and pluripotent inner cell mass (ICM) are understood to arise from ‘outside’ and ‘inside’ cells, respectively (Johnson and Ziomek, 1983; reviewed by Johnson and McConnell, 2004; Rossant and Tam, 2009).

Two models have been put forward to explain the cellular basis for the segregation of the TE and ICM lineages (reviewed by Yamanaka et al., 2006; Wennekamp et al., 2013). According to the ‘inside-outside’ model (Tarkowski and Wroblewska, 1967), blastomeres differentiate into TE or ICM depending on their position at the 16-cell stage or later. Cell fate is not determined by inherent differences in the cells but rather, inside and outside cells are thought to respond to differences in the mechanical or chemical stimuli they might be subjected to as a result of their position.

The ‘cell-polarity model’ suggests rather that the orientation of cleavage of polarised blastomeres at both the eight- and 16-cell stages determines the fate of their daughters, depending on the equal or unequal partitioning of lineage determinants distributed asymmetrically along the apicobasolateral axis of the mother blastomere (Johnson and Ziomek, 1981). In this view, polarised outside blastomeres can divide in two fundamentally different ways. In ‘symmetric’ divisions, the cleavage plane is aligned with the axis of polarity of the cell so the resulting daughters are both polar, remain outside and only commit to the TE fate at the 32-cell stage. In ‘asymmetric’ divisions, the cleavage plane is roughly orthogonal to the axis of polarity of the cell, resulting in daughters that are non-equivalent, since one inherits most or all of the apical domain and the other little or none. Here, the outer daughter remains polarised, having an apical face contributing to the outer surface of the morula, whereas the inner daughter is apolar and remains completely embedded within the morula. Apolar inside cells formed in this way give rise to the ICM.

The cell polarity model in general is widely accepted, as it provides a simple and straightforward explanation of how lineage decisions might be made. It is based on a series of elegant experiments that pushed the limits of the technology available at the time. However, most of the experiments per force relied on relatively invasive approaches requiring disaggregation of the embryo (Johnson and Ziomek, 1981; Ziomek et al., 1982). This is particularly a concern given that proteins, such as Yap, that are important in cell-fate specification at this stage (Nishioka et al., 2009), have also been implicated in transducing information about the cell’s mechanical environment (Dupont et al., 2011). As the mouse embryo is highly regulative, it can develop to give normal offspring even after experimental manipulation, but the observations made in such perturbed embryos might not be representative of ‘normal’ development but of ‘regulative’ or altered development. More recently, time-lapse microscopy has been used to study lineage allocation in unmanipulated mouse embryos (Bischoff et al., 2008; McDole et al., 2011). Both these studies, however, have been based on imaging and tracking only blastomere nuclei as a proxy for the entire blastomere. These studies have had to make deductions about whether blastomeres are ‘inside’ or ‘outside’ based on the proximity of nuclei to the surface of the embryo, rather than directly by visualising the blastomere surface.

In this report, we produce cellular resolution digital representations of embryonic development to test the cellular basis for lineage segregation. Our results indicate that there is considerable movement of outside cells to the inside and that the angle of division is less important than the position of a cell in lineage determination.

## RESULTS

### Time-lapse imaging of inner cell mass formation

**Fig. 1.**
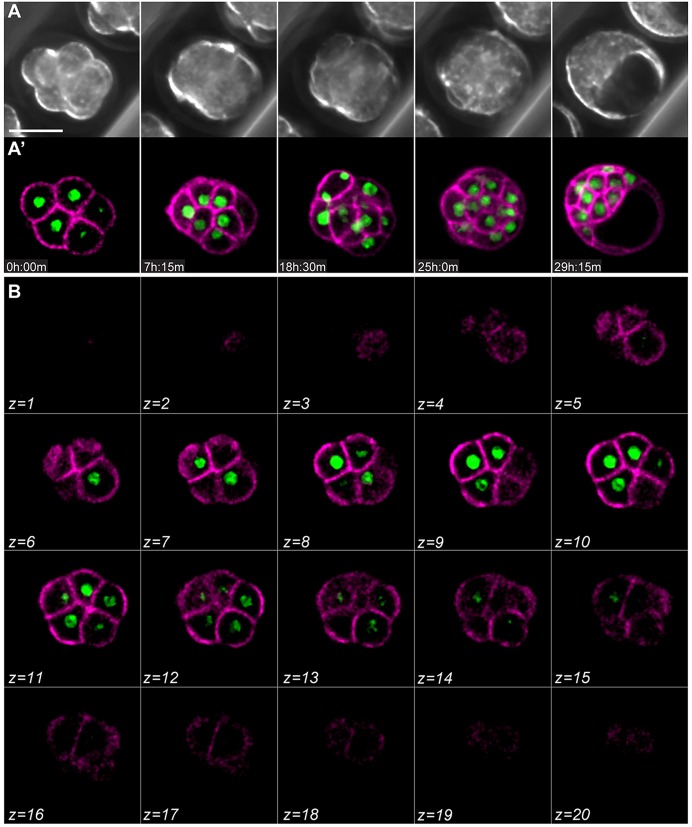
**4D time-lapse microscopy of blastocyst formation.** (A,A′) Time-lapse images of a CAG-TAG transgenic mouse embryo developing from morula to blastocyst. (B) Different focal planes of the same embryo, at a single time point. Nuclei are green (H2B-GFP) and plasma membranes are magenta (myr-TdTomato). Scale bar: 50 µm. Also see supplementary material Movies 1 and 2.

We performed time-lapse confocal microscopy on transgenic embryos in which the plasma membrane was visualised with membrane-TdTomato and the nucleus with H2B-GFP (Trichas et al., 2008). Embryos imaged from the eight- to 32-cell stage (Fig. 1A,B; supplementary material Movies 1 and 2) showed normal development from morula to blastocyst, over time scales typical for *in vitro* cultured embryos. To determine whether embryos suffered photodamage as a consequence of imaging, we transferred them into pseudopregnant recipients. Imaged embryos produced live-born offspring at similar frequencies to control embryos cultured in the microscope incubation chamber without imaging (supplementary material Table S1). Both males and females born from imaged embryos were fertile, indicating that imaging embryos under our conditions from the morula to early blastocyst stage does not cause any obvious damage to the soma or germline.

**Fig. 2.**
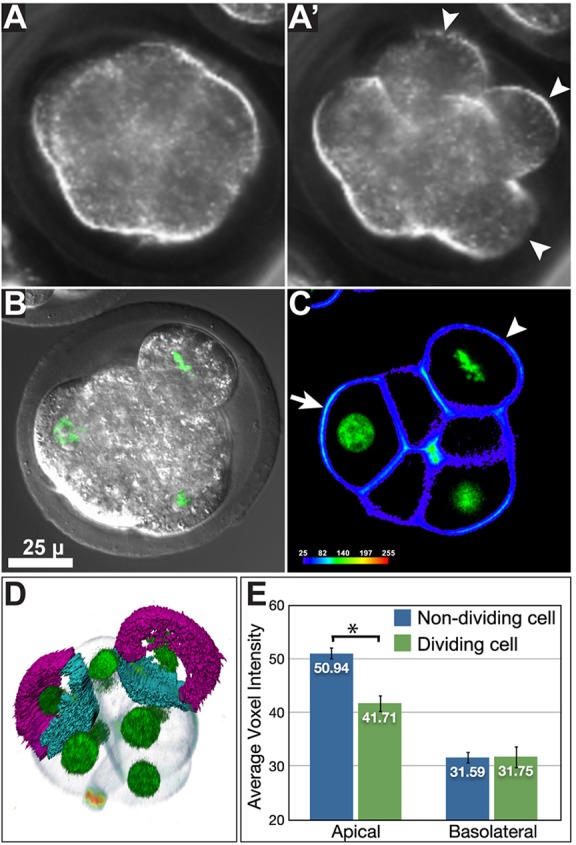
**Blastomeres in the compacted morula lose polarity during division.** (A,A′) Brightfield images of compacted morula undergoing cleavage division. Immediately prior to division, blastomeres round up and take a more superficial position in the embryo (arrowheads in A′). (B) Morula in the process of blastomere division, imaged immediately after isolation from the oviduct. As in embryos imaged during culture *in vitro*, the dividing blastomere rounds up and becomes superficial. (C) Dividing cells (metaphase chromosomes visible by H2B-GFP) have reduced apicobasolateral polarity, as shown by reduced apical signal of myr-TdTomato (arrowhead) in comparison to nondividing blastomeres (arrow). (D) Loss of polarity was quantified by volume segmentation of apical (magenta) and basolateral (cyan) domains of blastomeres. The basolateral domain of cells was considered the portion in contact with other cells. (E) Dividing blastomeres show significant reduction in apical TdTomato signal. *n*=10 dividing and 18 nondividing blastomeres from nine embryos. *P=0.00143 (Student's unpaired *t*-test).

Time-lapse data showed that morulae undergo a degree of decompaction during cell division events. Dividing blastomeres typically round up, and take on a more superficial position in the embryo, often appearing to almost be separate from the remainder of the embryo, which still appears compacted (Fig. 2A,A′). To determine if this behaviour is an artefact of embryo culture or imaging, we isolated 3.0 dpc morula and imaged them straight away, to catch them as they were undergoing cell division. We observed a similar decompaction of dividing blastomeres in noncultured embryos (Fig. 2B). TdTomato is localised to the plasma membrane by fusion to the membrane localisation domain of the Lyn intracellular kinase (Trichas et al., 2008). Such fusion proteins can be used as a readout of apicobasolateral polarity, as they are present at higher levels in the apical domain of polarised cells (Burtscher and Lickert, 2009). We compared average voxel intensity of TdTomato in the apical and basolateral domains of dividing and nondividing cells. When compared with nondividing cells, dividing cells showed a reduction in the ratio of apical to basolateral TdTomato, consistent with them losing a degree of apicobasolateral polarity during division (Fig. 2C-E).

### Digitising early embryonic development

**Fig. 3.**
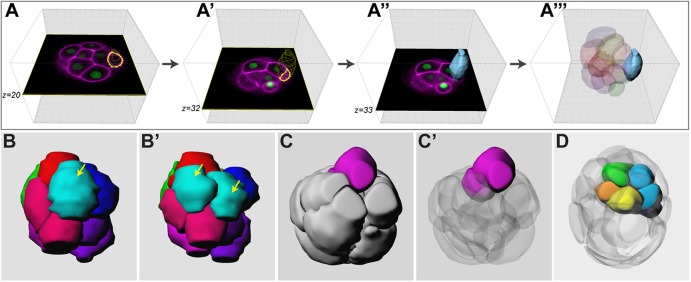
**Digitising mouse embryo development.** See supplementary material Movie 3 for an animation of how bitmap data were converted into a vector representation. (A-A‴) Individual blastomeres were manually outlined to create vector representations of the component cells of the embryo. Blastomeres can be tracked over time and through divisions, allowing one to map their fate. Once digitised in this way, the embryo can be viewed from any angle, specific lineages can be colour coded, made transparent, etc. (B,B′) Two consecutive time points from a digitised embryo showing a symmetrical division (yellow arrows). (C,C′) Two different renderings of the same time point showing the result of an asymmetric division. In C, only the outer sister is visible, but in C′ surrounding blastomeres have been rendered semi-transparent, so that the inside sister is also visible. (D) A representative blastocyst in which TE cells have been rendered semi-transparent, so as to reveal the ICM. Cells in the ICM are coloured on the basis of the blastomere from the eight-cell stage they derive from. Also see supplementary material Movie 4.

It is difficult to visually track the movement of individual cells or quantitatively analyse their behaviour in raw 4D image data. We therefore built 4D cellular resolution vector reconstructions of six embryos imaged from the eight- to 32-cell stage, by manually segmenting individual constituent blastomeres in each embryo (Fig. 3A; supplementary material Movie 3). Segmentation allowed us to convert each blastomere in the bitmap image volume into a vector representation. The time resolution of the image data was sufficient to track blastomeres (on the basis of position and morphology) from one time point to the next and to assign mother-daughter relationships during cell division and therefore, track lineage relationships. Cells at the 32-cell stage could be identified as ICM or TE by their relative position and morphology.

These cellular resolution vector representations of the developing embryo encapsulated information on not only the location, shape and movement of blastomeres, but also the fate of each blastomere from morula to blastocyst, so we call them ‘digital embryos’. As the display properties of individual blastomeres such as colour and transparency could now be manipulated at will, one could not only view the embryo from different angles, but also colour code specific lineages (Fig. 3B,C; supplementary material Movie 4) or make the outer cells ‘transparent’ to follow the formation of the ICM, which would otherwise be obscured by overlying cells (Fig. 3D; supplementary material Movie 4). The digital embryos are also amenable to interrogation for quantitative parameters of blastomeres such as volume, surface area and angle of division.

### Verification of segmentation accuracy and extracting quantitative information

One always has to balance high image quality (and the resultant energy load on the embryo) and minimally perturbed development, which better reflects normal *in utero* development. For our time-lapse experiments, we had to image embryos at relatively low resolution (128×128×20 pixels *x*, *y*, *z*). Embryos imaged at higher resolution appeared to develop normally during culture, but failed to produce viable offspring when transferred into recipients. To verify that the spatial resolution of the time-lapse data was sufficient for accurate segmentation, we imaged three embryos at a single time-point at the ‘low’ resolution used for time-lapse studies (128×128×20) as well as at ‘high’ resolution (512×512×40). The image volumes were then segmented independently by two different experimenters, blind to which low- and high-resolution volumes corresponded to each other. For all embryos, the blastomeres identified from the low-resolution image data were identical to those from the high-resolution image volumes. Furthermore, there was no statistically significant difference in surface area and volume between blastomeres from the two groups (supplementary material Fig. S2), suggesting that the resolution we used for time-lapse imaging was sufficient for accurate identification and segmentation of individual blastomeres.

We next developed custom perl and Mathematica scripts to extract key metrics pertaining to each blastomere, such as surface area, volume and centre of mass from the data files representing the digital embryos. These blastomere volume measurements were used in conjunction with visual inspection of the image data when making lineage assignments of dividing blastomeres, using the reasoning that the sum of volumes of daughter cells would be approximately equal to the volume of the mother cell.

### Fate of blastomeres as a function of angle of division

**Fig. 4.**
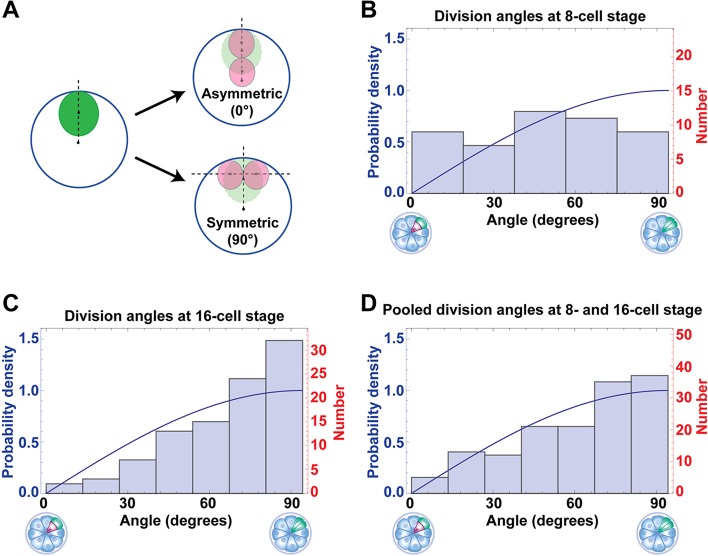
**Distribution of angles of division at the** **eight- and 16- cell stages.** (A) Cartoon depicting how the angle of division was calculated (see Materials and Methods for details). The large blue circle represents the entire embryo, the green and light-green ovals the mother blastomere and the pink circles the daughter blastomeres. (B,C) Histogram of angles of division at the eight-cell and 16-cell stage. (D) Histogram of pooled angles of division for both the eight- and 16-cell stages. The blue curve plots sin(θ), the expected isotropic distribution of angles. At the eight-cell stage, there is a slight trend towards more asymmetric divisions than expected (*P*=0.0602, χ^2^ test). At the 16-cell stage, significantly more symmetrical divisions than expected are seen (*P*=0.012, χ^2^ test).

Divisions can be considered symmetric or asymmetric on the basis of the angle of division or the extent to which the resulting daughter cells are exposed to the outside of the embryo. We first considered blastomere divisions in terms of the angle of division. An asymmetric division can be defined as one in which the line passing through the centres of mass of the two daughters is roughly parallel to the line passing through the centres of mass of the mother blastomere and embryo, so that the angle subtended by them is 0°. In a symmetric division, these two lines would be perpendicular to each other, subtending 90° (Fig. 4A). The histogram of angles of division between the eight- and 16-cell stage shows a trend towards more asymmetric divisions than would be expected from an isotropic distribution [described by the function sin(θ); see theoretical methods in the supplementary material for explanation]. Interestingly, divisions between the 16- to 32-cell stage showed the opposite bias, with significantly more symmetric divisions than might be expected from an isotropic distribution. Pooling division angles at the eight- and 16- cell stages gives a distribution that more closely fits sin(θ) (Fig. 4B-D).

**Fig. 5.**
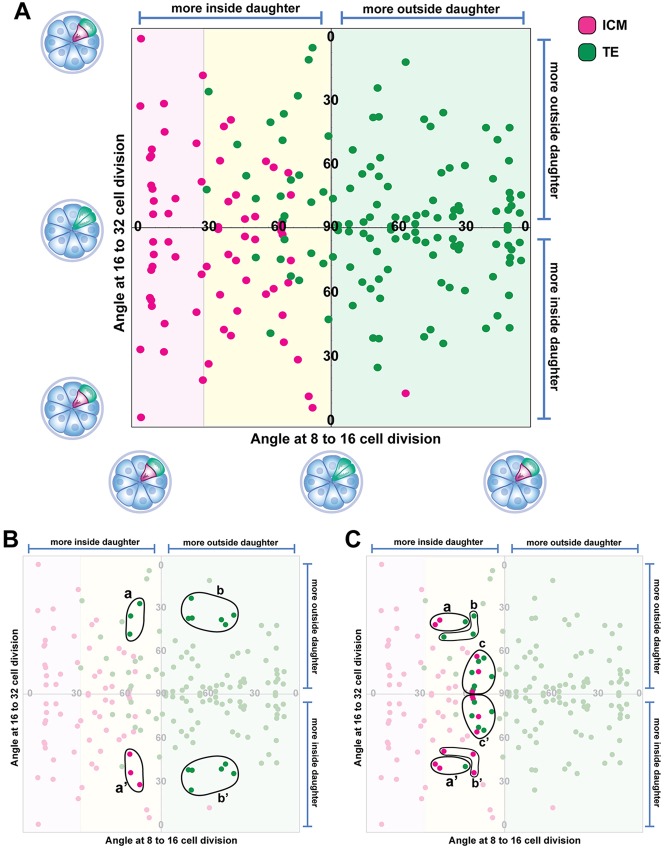
**Plot relating fate of blastomeres to the history of their angles of division.** See supplementary material Movie 5 for an animation explaining how this plot is derived. (A) Each cell represents a cell from 32-cell stage embryos. Their position on the plot is determined by the angle of division of their mother and grandmother blastomeres and the relative proximity of the resulting daughters to the centre of mass of the embryo. (B,C) Same plot as in A, with specific groups of cells highlighted to illustrate that similar angles of division can give very different outcomes (see text for details).

As outlined above, 0° represents a ‘perfectly’ asymmetric division, whereas 90° represents a ‘perfectly’ symmetric division. In reality, division angles are distributed somewhere between these two extremes (Fig. 4B,C) and the majority are ‘oblique’ angles. To test the relationship between the angle of division and subsequent fate, one could set arbitrary ranges of division angles for example, 0°-30°, 31°-60° and 61°-90° as being asymmetric, oblique and symmetric, respectively. This, however, can give different results depending on the specific ranges defined. We therefore visualised the data as a graph that plots each cell of a 32-cell stage blastocyst in relation to the history of division angles of its mother (at the 16-cell stage) and grandmother (at the eight-cell stage), so one can more easily see the continuum of behaviour with division angles (Fig. 5A). See supplementary material Movie 5 for an animation showing how the graph is plotted. Each cell at the 32-cell stage was plotted as a dot with the colour denoting ICM or TE. The position of the dot represents the history of division angles of the cell's mother (in the 16-cell morula) on the *y*-axis and grandmother (in the eight-cell morula) along the *x*-axis. The origin is at the centre of the plot and for any division event, the daughter more on the outside of the embryo (as determined by the location of the centre of mass of the blastomere with respect to the centre of mass of the whole embryo) is plotted above or to the right of the origin, whereas the more centrally located daughter is plotted below or to the left of the origin. Therefore, dots show mirror-image symmetry about the *x*-axis, as sisters resulting from the division of 16-cell blastomeres will have the same value for angle of division, but will be plotted towards the top or bottom of the chart depending on whether they were farther or closer from the centre of the embryo (supplementary material Movie 5).

If ICM cells are specified as a result of asymmetric divisions at the eight- to 16-cell and 16- to 32-cell rounds of divisions, one would expect ICM cells to be present only along the far left and bottom margins of the plot. TE cell would be expected to cluster to the top right quadrant. The observed plot was very different from this expected pattern in several ways.

First, there was a conspicuous absence of ICM cells at the bottom right of the plot – those that would have been expected to form as a result of asymmetric divisions of blastomeres of the 16-cell morula.

Second, there were a relatively large number of TE cells in the bottom-right quadrant. These are the ‘inside’ daughters of reasonably asymmetric (≤45°) divisions at the 16- to 32-cell stage that nevertheless went on to become TE rather than ICM. Interestingly, similar division angles produced ICM cells if the mother was the slightly ‘more inside’ daughter of a symmetrical (≥60°) division at the eight- to 16-cell stage (the bottom-left quadrant) (Fig. 5B, compare regions a,a′ and b,b′).

Third, there was only little clustering of cells along the *y*-axis, and this was restricted to the two left-hand quadrants, representing the progeny of ‘more inside’ daughters of eight- to 16-cell divisions. But even here, the clustering was imperfect, with considerable overlap between angles of division that give symmetric (Fig. 5C, regions a,a′) and asymmetric fates (Fig. 5C, regions b,b′). This suggests that the angle of division at the 16-cell stage by itself has limited predictive power.

Finally, there is clear clustering of cells along the *x*-axis, suggesting that the orientation of division at the eight-cell stage has reasonable predicative power (Fig. 5A). When a blastomere in the eight-cell morula divides, regardless of whether it is asymmetrical or largely symmetrical, the daughter slightly more to the outside of the embryo appears to be fated to contribute exclusively to the TE, even if the subsequent division is highly asymmetric (Fig. 5A, green shaded region). When a blastomere in the eight-cell morula divides strongly asymmetrically (for example ≤30°), the inside daughter is fated to contribute exclusively to the ICM (Fig. 5A, pink shaded region). The range of angles that give inside cells that predominantly form ICM (pink shaded region from 0° to 30°, Fig. 5A) is considerably narrower than the range of angles giving outside cells that predominantly form TE (green shaded region from 90° to 0°, Fig. 5A). It is the ‘more inside’ daughters from largely symmetric divisions at the eight-cell stage that have a somewhat mixed fate, contributing to both TE and ICM (Fig. 5A, yellow shaded region). Strongly asymmetric division (≤30°) of these outside cells tends to result in the inner daughter becoming ICM and the outer daughter becoming TE (Fig. 5A). However, at less clearly asymmetrical angles of division (≥60°), these outside cells can give rise not only to two TE cells, but surprisingly, also to two ICM cells (Fig. 5C, regions c,c′), pointing to factors other than division angle influencing lineage allocation in this group of cells.

### Fate of ‘inside’ and ‘outside’ cells

We next considered blastomere divisions on the basis of the proportion of the daughters’ surface exposed to the outside. We defined ‘surface exposure’ as the ratio of external area to total surface area and calculated this for each blastomere over time. An asymmetric divisions would be expected to produce one daughter with low or negligible surface exposure (the inside blastomere) and one with considerable surface exposure (the outside blastomere).

**Fig. 6.**
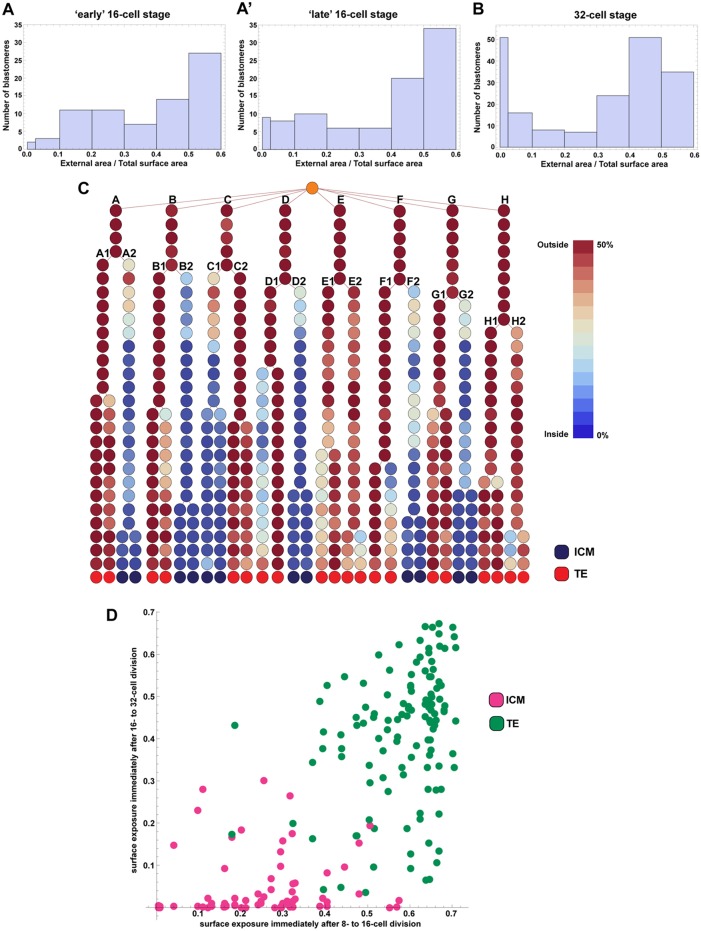
**Fate of inside and outside blastomeres.** (A,A′) Histogram of surface exposure at the earliest and latest 16-cell stage. (B) Histogram of surface exposure at the 32-cell stage. Note that the bins are not all the same size. (C) Lineage tree of a representative embryo showing development from the eight- to 32-cell stage. More details on this embryo in supplementary material Fig. S8. Blastomeres are coloured according to their surface exposure. The outside sister of asymmetric divisions at the eight-cell stage (for example, blastomeres B1 and E1) are fated to contribute to only the TE, even if, when they divide at the 16-cell stage, they do so asymmetrically to give a daughter that is initially mostly on the inside. In digitising embryo development, as not all time points were segmented, the time interval in the lineage tree between one row and the next is not constant.

We plotted histograms of blastomere surface exposure at the 16- and 32-cell stages (Fig. 6A,A′,B). We considered the 16-cell stage at the earliest time after the formation of 16 blastomeres, as well as the last time point before the next round of division started, which we refer to as early and late 16-cell stage, respectively. At the early 16-cell stage, embryos had almost no cells with negligible surface exposure. The average number of inside cells was 0.33, which is much lower than the average of five to six reported by some authors (Kimber et al., 1982; Fleming, 1987) but in agreement with averages ranging from 0.17 to 1.70 reported by others (Graham and Lehtonen, 1979; Dietrich and Hiiragi, 2007; Fujimori et al., 2009; Shipley et al., 2009). However, by the late 16-cell stage the number of inside cells had increased to an average of 1.5 cells per embryo, showing that inside cells can arise independent of cell division. At the 32-cell stage, embryos had an average of 8.5 inside cells (Fig. 6B).

Using custom perl and Mathematica scripts, we generated a lineage tree for each digital embryo in which we colour coded each blastomere on the basis of its surface exposure at that time point (Fig. 6C; supplementary material Figs S3-S8, panels A). Such a representation reveals that during the stages considered, blastomeres are extremely dynamic in their relative position within the embryo with respect to the outer surface. Even the most ‘asymmetric’ divisions do not produce cells completely embedded inside the embryo without any exposure to the outside. Rather, the surface exposure of daughters immediately after division shows a continuous rather than bimodal distribution of values (Fig. 6A,A′). Blastomeres do not necessarily maintain their relative positions from the time of division but move towards or away from the surface of the embryo, changing their surface exposure over time (for example, blastomere A2 in Fig. 6C). Plotting the radial movement of cells toward or away from the centre of the embryo on these lineages indicates that changes in surface exposure broadly correspond to radial movement of cells (supplementary material Figs S3-S8, panels B). Plots of overall relative movement of cells show that blastomere movement corresponded to the waves of division events (supplementary material Figs S3-S8, panels C) suggesting that cell rearrangements might occur passively, as a result of ‘jostling’ during division events.

Some lineages are consistent with the hypothesis that relatively internal cells of an asymmetric division give rise to ICM. For example in Fig. 6C, at the eight-cell stage, blastomere B divides relatively asymmetrically and the daughter more to the outside (B1) gives rise to TE, whereas the other (B2) gives rise to ICM. However, most divisions are not consistent with this straightforward view, particularly at the 16-cell stage, where we find several instances of what might be considered asymmetrical divisions, in which one daughter is distinctly more to the interior than the other. Nevertheless, both daughters give rise to TE. For example, blastomere F1 divides relatively asymmetrically, but still produces two TE cells.

To determine the relationship between surface exposure and fate, we plotted each cell at the 32-cell stage on the basis of its surface exposure at formation against the surface exposure of its mother immediately after formation (Fig. 6D). The plot shows a clear segregation of ICM and TE cells, but with some overlap between the two. Cells that have a surface exposure greater than 0.6 when they form at the 16-cell stage appear to only be able to give rise to TE cells in the next round of division, even when they have daughters with surface exposures as low as 0.1. However, if the mother at the 16-cell stage had a surface exposure in the range of 0.4 to 0.5, and her daughters at the 32-cell stage had surface exposures in the range of 0.05 to 0.2, there is a great deal of heterogeneity in fate, with both TE and ICM cells being formed.

## DISCUSSION

### Asymmetric and symmetric divisions versus subsequent fate

We have considered symmetric versus asymmetric divisions based on the surface exposure and angle of division of blastomeres. By both these criteria, our analysis of cell division and fate in digital embryos reveals behaviour that runs counter to expectations from the cell polarity model (Johnson and Ziomek, 1981).

The cell polarity model suggests that the angle of division of ‘outside’ cells at both the eight- and 16-cell stages determines the fate of daughter cells. We find that while the angle of division at the eight-cell stage is predictive of subsequent fate, that at the 16-cell stage is much less so. Moreover, although the angle of division at the eight-cell stage is predictive, it bears a more complicated relationship to the subsequent fate of cells than suggested by the cell polarity model. According to the model one would expect that when a blastomere divides asymmetrically at the eight-cell stage, it is only the resulting inside cell that is committed, contributing only to ICM, whereas the outside cell remains uncommitted, able to give rise in the next round of division to either ICM or TE. However, we find that although it is true that inside daughters of asymmetric divisions at this stage contribute only to ICM, the outside daughters of such divisions are also restricted, contributing only to TE in the subsequent round of division. Even in divisions that could be classified as symmetrical, the ‘slightly more’ outside daughter inevitably contributes only to the TE (barring one exception at the 16-cell stage that gives the single ICM cell in the bottom-right quadrant in Fig. 5A). It is the slightly more inside daughter that retains the potential to give rise to either ICM or TE in the next round of division. Similar behaviour is revealed when considering surface exposure, where an outside cell at the 16-cell stage with an exposure greater than 0.6 can contribute only to the TE, regardless of the surface exposure of its daughters immediately upon division (Fig. 6D).

**Fig. 7.**
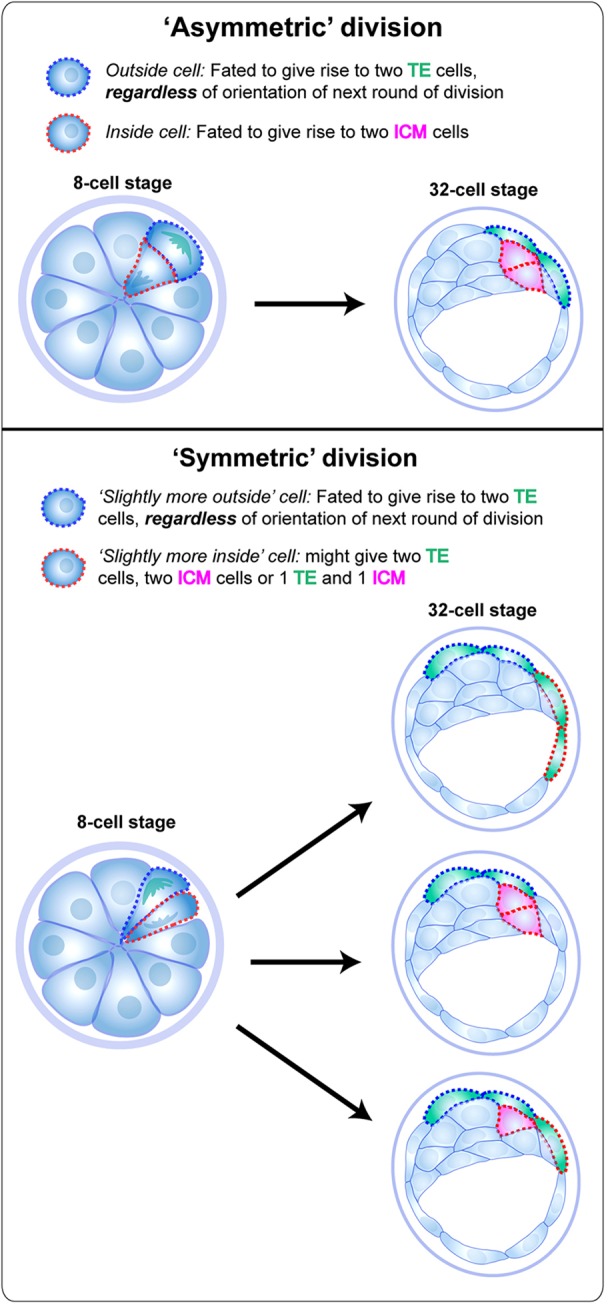
**Graphical summary of the relationship between angles of division and fate.** Depiction of the possible outcomes at the 32-cell stage of asymmetric (top panel) and symmetric (bottom panel) division of a blastomere at the eight-cell stage.

Our data therefore support the argument that as early as the 16-cell stage, outside cells are not all equivalent. Some contribute to both the ICM and TE (the slightly more inside sisters of near symmetrical divisions), whereas others are fated to contribute only to the TE (the slightly more outside sisters of near symmetrical divisions, and the outside sisters of asymmetrical divisions) (Fig. 7). In this sense, there are no truly ‘symmetrical’ divisions at the eight-cell stage, as one essentially never observes two daughters both having the potential to give rise to TE as well as ICM.

We therefore suggest that at the 16-cell stage, it is not the ‘inside’ cell that is committing to become ICM with the outside cell retaining the potential to give rise to either ICM or TE in the subsequent round of division, but rather the outside cell whose fate is being restricted to forming only TE (Fig. 7). This view is consistent with the ICM (formed from inside cells) being the lineage that retains greater potential than the TE. It is also consistent with the fact that in embryos in which the TE is immunosurgically ablated, the newly exposed ICM is able to differentiate into TE (Handyside, 1978).

At the 16-cell stage, among those outside blastomeres capable of contributing to both ICM and TE, similar angles of division can give rise to very different outcomes, with both daughters sometimes forming either TE or ICM, and at other times one forming TE and the other ICM. This does not support the notion of angle of division determining cell fate. In general, however, when a division results in two cells of different fate, it is the daughter that is more to the outside that contributes to the TE, whereas the more inside daughter forms the ICM. Similarly, cells with a lower surface exposure are more likely to give rise to ICM.

Asymmetric divisions at both the eight- and 16-cell stages would place one daughter more to the outside, with a greater proportion of its limiting membrane not in contact with surrounding blastomeres. ‘Symmetric’ divisions at the eight-cell stage give rise to two outside daughters that nevertheless have very different potential, based on which is more to the outside. Furthermore, very similar symmetrical divisions of outside cells at the 16-cell stage can have different outcomes, sometimes resulting in two TE cells, but also sometimes resulting in two ICM cells (Fig. 5C, regions c,c′). Cells can move inwards at the 16-cell stage (Fig. 6A,A′,C), possibly accounting for the limited predictive value of the angle of division in relation to fate. Such movement might also explain the contrasting fates of blastomeres with broadly similar surface exposure (between 0.05 and 0.2 at the 32-cell stage, Fig. 6D). This suggests that the position of a cell deeper or more superficial in the embryo might be as important as the angle of division, if not more, in determining the fate of the cell. Nishioka and colleagues have shown that the extent of contact with surrounding cells might lead to the specification of cell fate (Nishioka et al., 2009). More recently, the same group has shown the importance of cell polarity in correct Yap-dependent lineage specification (Hirate et al., 2013). Taken together, this is consistent with the inside-outside model, with cells using extent of cell contact or polarity (or a combination of both) to read-out their position in the embryo and accordingly activate or inactivate Yap-dependent Tead4 activity.

### Anisotropic distribution of angles of division

It has been suggested that ‘inside’ cells produced by the asymmetric division of eight-cell blastomeres might preferentially contribute to the epiblast, whereas inside cells produced by asymmetric division of 16-cell blastomeres preferentially contribute to the primitive endoderm (Yamanaka et al., 2006). Subsequent reports testing this model have been conflicting, either disproving (Yamanaka et al., 2010) or supporting (Morris et al., 2010) it. Our data show that there are significantly fewer asymmetric divisions at the 16-cell stage than might be expected if the angle of division was random (Fig. 4C), arguing against this model. Our results differ from the findings of Morris et al. (2010), possibly because of the different imaging approach they take, which is based on approximations of cell position derived from following cell nuclei. It is also possible that differences among mouse strains might account for some of the differences observed.

There continues to be debate in the field regarding whether the development and fate of blastomeres is influenced by events at or before fertilisation (Gardner, 2005; Zernicka-Goetz, 2006), as opposed to being ‘stochastic’ (Marikawa and Alarcon, 2009; Wennekamp and Hiiragi, 2012). Our data do not speak to whether this deviation from the isotropic distribution is driven by an active determinant within a specific subset of blastomeres or by some indirect mechanism acting in a more stochastic manner, such as degree of contact with surrounding cells (Graham and Deussen, 1978; Nishioka et al., 2009) or some other geometrical attributes of blastomeres.

### Cellular resolution digital representation of blastocyst development

When imaging cultured embryos, it is important to carefully control for photodamage. We have demonstrated that the embryos we imaged for constructing our digital embryos were able to give rise to fertile adults. Notably, when we imaged embryos for longer durations, starting from the four-cell stage or continuing up to the 64-cell stage, although they appeared to develop normally morphologically, they did not give rise to pups when transferred into recipients, presumably as a result of the embryos suffering damage that was not morphologically evident during imaging. Similarly, when we imaged embryos at somewhat higher resolution or laser power, we again achieved what appeared to be normal development during the period of imaging, but were unable to recover live-born embryos from transfers. We have previously observed similar effects when imaging mouse embryos at longer wavelengths (Watanabe et al., 2010), highlighting the importance of controls that test the viability of imaged embryos by determining if they develop fully to term.

In order to minimise photodamage, we had to limit the quality and resolution (both spatial and temporal) of embryo imaging. This meant our study had to be restricted to morula to blastocyst stage development, preventing us from addressing some very interesting questions. Advances in light-sheet-based microscopes (Keller et al., 2008; Huisken and Stainier, 2009; Tomer et al., 2012), which cause considerably less photodamage than laser scanning confocal microscopes, should allow us in future to capture much higher quality recordings of embryo development without compromising viability. Such imaging approaches generate vast quantities of image data and parallel advances in automated segmentation, and tracking algorithms (Tomer et al., 2012) are vital in analysing the data generated. Although manual segmentation is considered the gold standard and is used to provide the ‘ground-truth’ against which automated algorithms are compared, it is extremely time-consuming, imposing constraints on the number of embryos that can practically be digitised and limiting the power of quantitative approaches.

By segmenting cell outlines, we were able to examine more accurately the contribution of angle of division to the fate of daughter blastomeres (Fig. 5) and more importantly, visualise the dynamic nature of blastomeres with regard to the proportion of their plasma membrane that is in contact with other blastomeres (Fig. 6). This approach was pioneered by Tassy et al. (2006) on fixed ascidian embryos that show determinate development. We have extended this approach to living embryos and into the additional dimension of time, to study the regulative development of the mouse ICM. Future improvements in imaging should enable us to generate large libraries of digital mouse embryos from fertilisation to implantation that can be interrogated for quantitative information and population behaviour. In combination with powerful modelling approaches (Shipley et al., 2009; Wennekamp et al., 2013), this will allow us to better understand the cellular basis for lineage segregation and axis formation in embryos that show regulative development.

## MATERIALS AND METHODS

### Embryo isolation and culture

Embryos were obtained through natural matings of heterozygous transgenic CAG-TAG1 males (Trichas et al., 2008) with wild-type CD1 females (Charles River). Noon of the day when the vaginal plug was found was considered 0.5 days post coitum (dpc). Embryos were isolated from the oviduct at 2.5 dpc in M2 media (Sigma) and then held in KSOM (Millipore catalogue number MR-020P-5F) before setting up for culture.

### Confocal time-lapse imaging

Embryos were cultured in glass-bottom dishes (MatTek Corporation; P35G-1.0-14-C) in a drop of KSOM (supplemented with sodium pyruvate, essential and nonessential amino acids) pre-equilibrated overnight at 37°C in 5% CO_2_. Culture medium was overlaid with pre-equilibrated mineral oil (Sigma-Aldrich; M8410) to prevent evaporation. Embryos were imaged on a Zeiss LSM 710 confocal microscope equipped with an environmental control chamber, using a 40×1.2 NA water immersion objective. EGFP was excited at 488 nm and TdTomato at 560 nm. To prevent embryos from drifting out of the field of view, their movement was restricted using hand-pulled thin glass filaments secured with vacuum grease (supplementary material Fig. S1). We collected 20 focal planes spanning the entire embryo at 15 min intervals. Embryos were imaged for up to 30 h until they reached the blastocyst stage. The excitation laser power was kept at the lowest possible (560 nm at 0.3%; 488 nm at 0.2%) and images were taken at an in-plane resolution of 128×128 pixels. We empirically determined imaging parameters suitable for normal development (as assessed by transfer into recipients) by starting at high image quality levels (512×512×40 pixels, laser power of ∼3%) and then worked our way down to lower resolutions until we obtained satisfactory viability. We disabled image averaging to further reduce pixel dwell time.

### Segmentation of confocal data

Imaris v6.3 (Bitplane) software was used for manual segmentation and lineage tracking of blastomeres. Cell outlines were drawn using a Wacom Cintique 21UX tablet display to trace the plasma membrane of each blastomere and create a 3D reconstruction using the ‘contour surface’ function (Fig. 3A; supplementary material Movie 3). Each cell was given a unique ID, colour coded and tracked over time. Only those time points in which blastomeres divided or showed considerable movement were segmented. The time resolution of the image data was sufficient to unambiguously track cells over time by comparing cell shape and position at different time points. When a cell divided, the daughter cells were assigned the same colour and ID code. Division of cells was confirmed by an independent second observer and by visualising the anaphase chromosomes at the previous time point. Imaris files of segmented embryos will be made available on request.

### Calculating blastomere surface area and angle of division

Custom perl scripts were written to calculate parameters such as blastomere surface area, volume and angle of division. The surface of each blastomere in the digitised embryo is represented as a meshwork of triangles. The total surface area of a blastomere can be calculated by summing the areas of the constituent triangles. To determine the ‘external’ surface area of a blastomere (the portion of surface area of the cell that is on the outside of the embryo), we considered each constituent triangle, fired a ray along its outward normal and asked whether it intersected any other triangles (of any of the blastomeres of the embryo) before it reached infinity. If it did not, the triangle was labelled as external, and conversely if it did, it was labelled internal.

The angle of division was measured by looking at the angle between the line from the centre of the mother cell and the centre of the embryo in the time point before division, and the line between the centres of the two daughter cells in the following time point 15 min later. The latter line is essentially normal to the cleavage plane, so a large angle between these two lines would indicate a radial cleavage, leaving behind symmetric daughters, whereas a small angle would indicate a tangential cleavage, leaving an inner and an outer daughter cell (Fig. 4A).

## Supplementary Material

Supplementary Material
