# Supplementary material for: Limited predictive value of blastomere angle of division in trophectoderm and inner cell mass specification
Source: Development. 2014 Jun;141(11):2279–88. doi: 10.1242/dev.103267 (PMC4034423; doi:10.1242/dev.103267)
Supplement: Supplementary Material [file supp_141_11_2279__index.html]

Limited predictive value of blastomere angle of division in trophectoderm and inner cell mass specification — Supplementary Material 

# Limited predictive value of blastomere angle of division in trophectoderm and inner cell mass specification

## DEV103267 Supplementary Material

**Files in this Data Supplement:**

- **Supplementary Material**
