# Supplementary material for: Limited predictive value of blastomere angle of division in trophectoderm and inner cell mass specification
Source: Development. 2014 Jun;141(11):2279–88. doi: 10.1242/dev.103267 (PMC4034423; doi:10.1242/dev.103267)
Supplement: Supplementary Material [file supp_141.11.2279_DEV103267.pdf]

Theoretical Supplementary Information

Origin of the sin(θ) distribution

One might expect that an isotropic distribution of divisions would correspond to every division angle being equally likely and hence a uniform distribution as a function of θ. This is true in two dimensions, but not in three dimensions. Imagine the embryo as a globe with an equator (0°), north (90°N) and south poles (90°S). We then extend many lines from the surface of this sphere to its centre. If these lines are isotropically distributed, they will fall evenly over the surface of the sphere. However, if we look at the angle these lines make with the north-south axis, many more of them will lie between 1° and 2° than between 89° and 90° because much more of the surface of the globe lies between 1° and 2° (which is near the equator) than between 89° and 90° degrees (which is near the pole). This effect gives rise to the Sin(θ) distribution for isotropically distributed angles in three dimensions. In the histograms in Figure 5, the specific relationship between the distribution and the angles is reversed (higher frequency expected at higher angles) because our line of reference (0°) corresponds to the 'north-south axis' created by the line passing through the centres of mass of the mother cell and the embryo which in the example above of a globe is 90°. A more technical description of the derivation of this distribution follows.

We imagine a sphere of radius one, with a z-axis protruding from the center of the sphere in a vertical direction. We then add a line from the origin to the edge of the sphere, making an angle θ with the z axis, as sketched below.

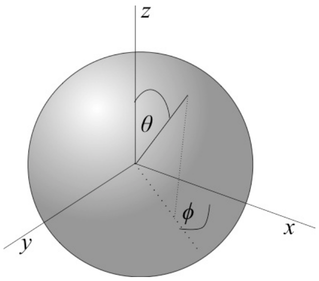

If the line we have added is equally likely to point in any direction, it is equally likely to touch the sphere at any point. Consequently, the probability a randomly generated line makes an angle θ that lies in the range $\theta 1<\theta< \theta 1+d\theta 1$ (where $d\theta 1$is a very small increment) with the z-axis is simply proportional to the fraction of the area of the sphere that lies in this range. The slither of sphere with these values of θ is sketched below.

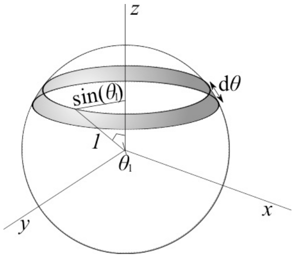

This thin slither of sphere can be unwrapped to form a strip approximated (exactly in the limit of small dθ1) by a rectangle with dimensions dθ1 and 2 π sin(θ1), giving it a total area of 2 π sin(θ1) dθ1. Since the whole sphere has area 4 π, this corresponds to a fraction of the surface of the sphere of 2 π sin(θ1) dθ1/(4 π)=(1/2) sin(θ1) dθ1, so a the probability a line drawn at random makes an angle θ1<θ< θ1+dθ1 with the z axis is P(θ1)dθ1 =(1/2) sin(θ1) dθ1, the sin(θ) distribution.

In terms of cell division, the above sphere should be thought of as embedded in the mother cell, with the z axis pointing out from the center of the embryo, through the center of the mother, to the outside. The line that is added is the line between the centers of the two daughter cells, translated to run exactly through the origin of the sphere. Consequently, in our case there is no distinction between division angles of θ and π- θ, since if one daughter cell is at θ the other is at π- θ. Thus, we are effectively only interested in the upper half-sphere, which has area 2 π, so we have P(θ1)dθ1 =sin(θ1) dθ1.

Definition of the center of each cell

For calculating quantities such as cell velocity and division angles we have to assign a point position to each cell. There are several ways one might do this. To assign this position we took the three-dimensional surface corresponding to the cell membrane and imagined filling it with a uniformly dense substance then calculated where the center of mass of such an object would lie. To do this calculation, we imagined a plane running though the cell, and broke the volume of the cell into long thin prismatic objects made by sweeping the triangles of the cells surface down to the plane (along the planes normal). Each of these basic objects has a known volume (i.e. ``mass'') m_i and a known position of its center of mass $\boldsymbol{r}_i$. The center of mass of the complex shaped cell, $\boldsymbol{R}$, is then given as

$$\boldsymbol{R} = \frac{\sum m_i \boldsymbol{r}_i}{\sum m_i}.$$

Definition of the velocity of each cell

To calculate the velocity of a cell we could have simply divided the distance between the center of the cell in two adjacent time steps by the time elapsed. However, sometimes the whole embryo rotates, which gives all the cells a velocity but doesn't

imply any internal change in the embryo. To eliminate this problem, we first rotated the embryo in the second frame until the centers of the cells were as close as possible to the centers of the same cells in the previous time step (specifically minimum total squared distance between cells, implemented using mathematica's NMinnimize function) then calculated the cell velocities as above. To calculate the radial velocity of a cell we simply projected the velocity vector onto the line connecting the CoM of the cell to the CoM of the embryo.

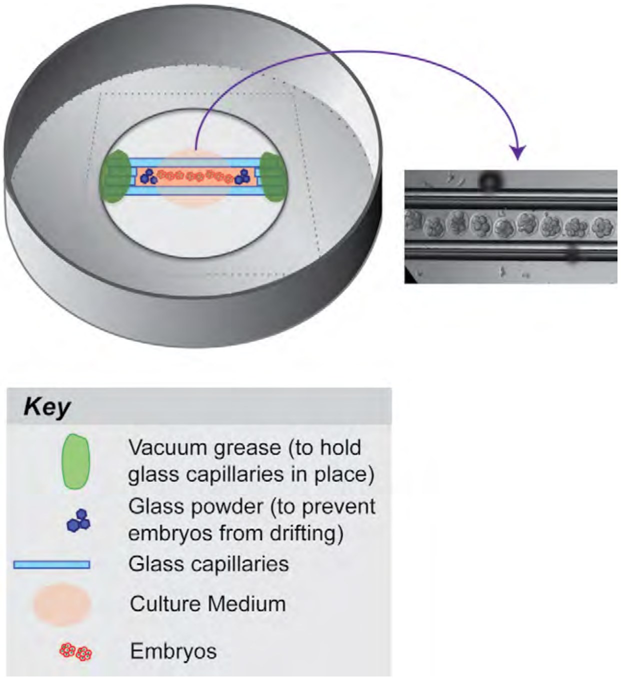

Supplementary Figure S1. Embryo culture dish for time-lapse imaging. Embryos were placed in a Matek dish, between two hand-pulled glass filaments held down with vacuum grease.

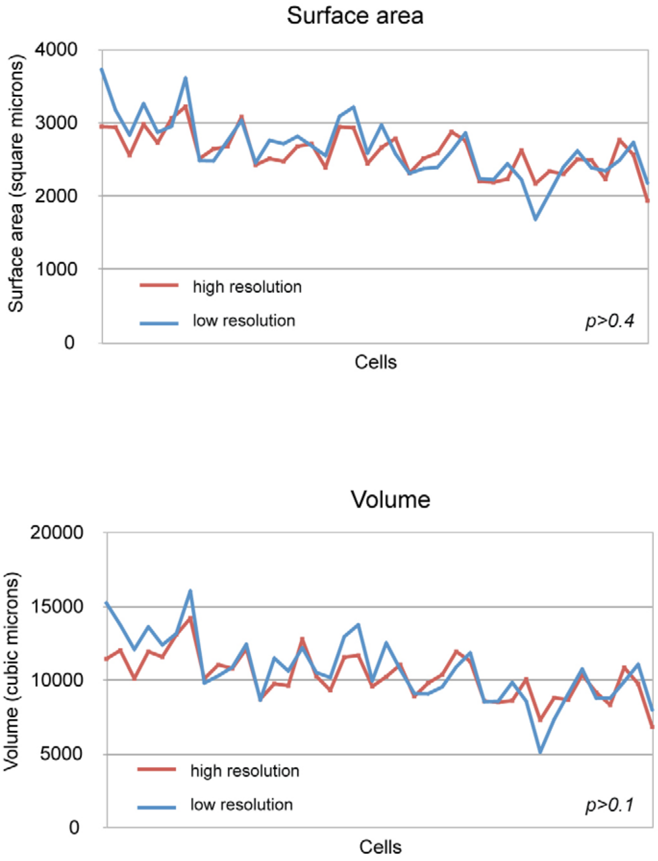

Supplementary Figure S2. Comparison of volume and surface area of blastomeres segmented from high and low resolution data. There is no significant difference between the values obtained with these two approaches.

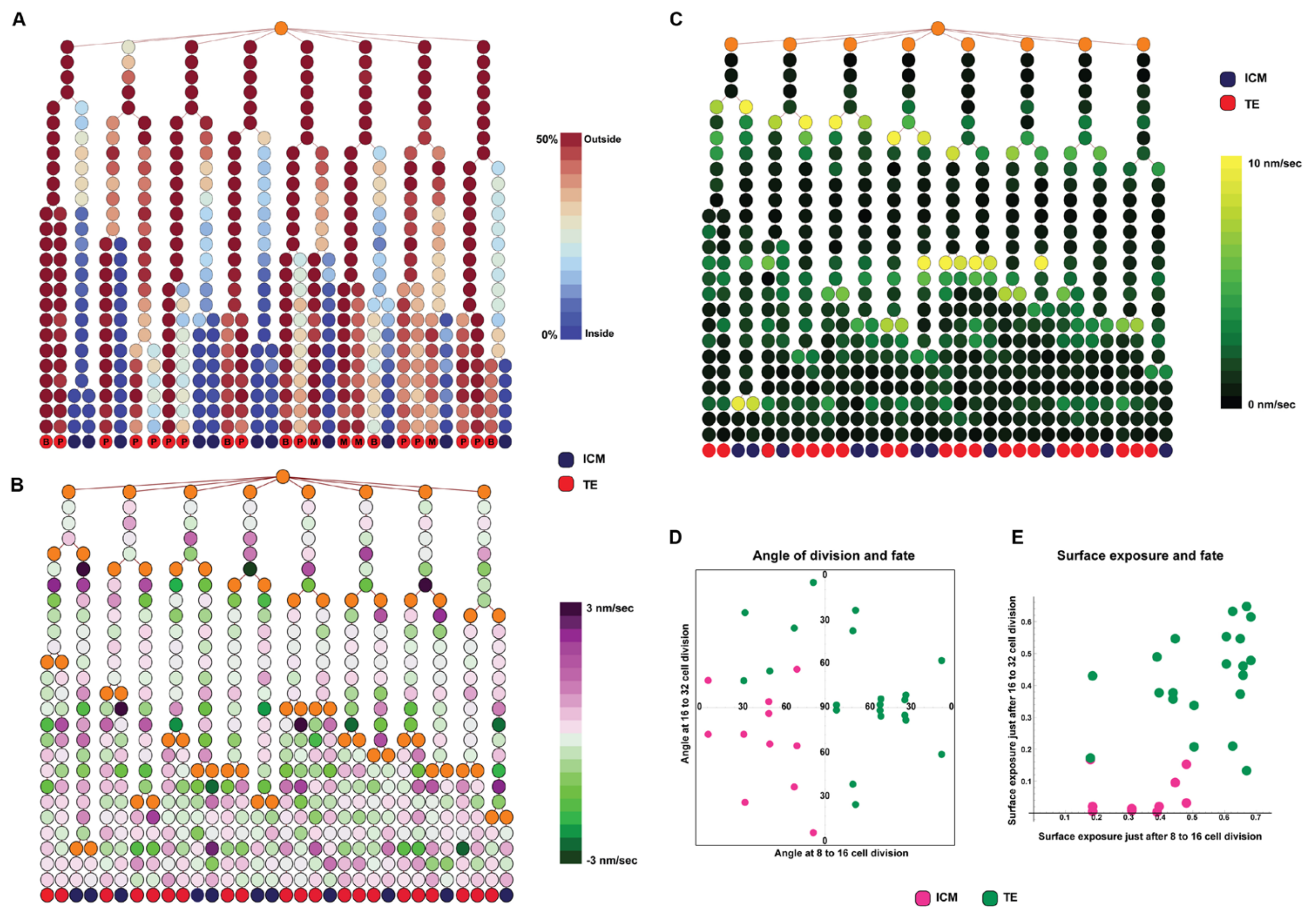

Supplementary Figure S3

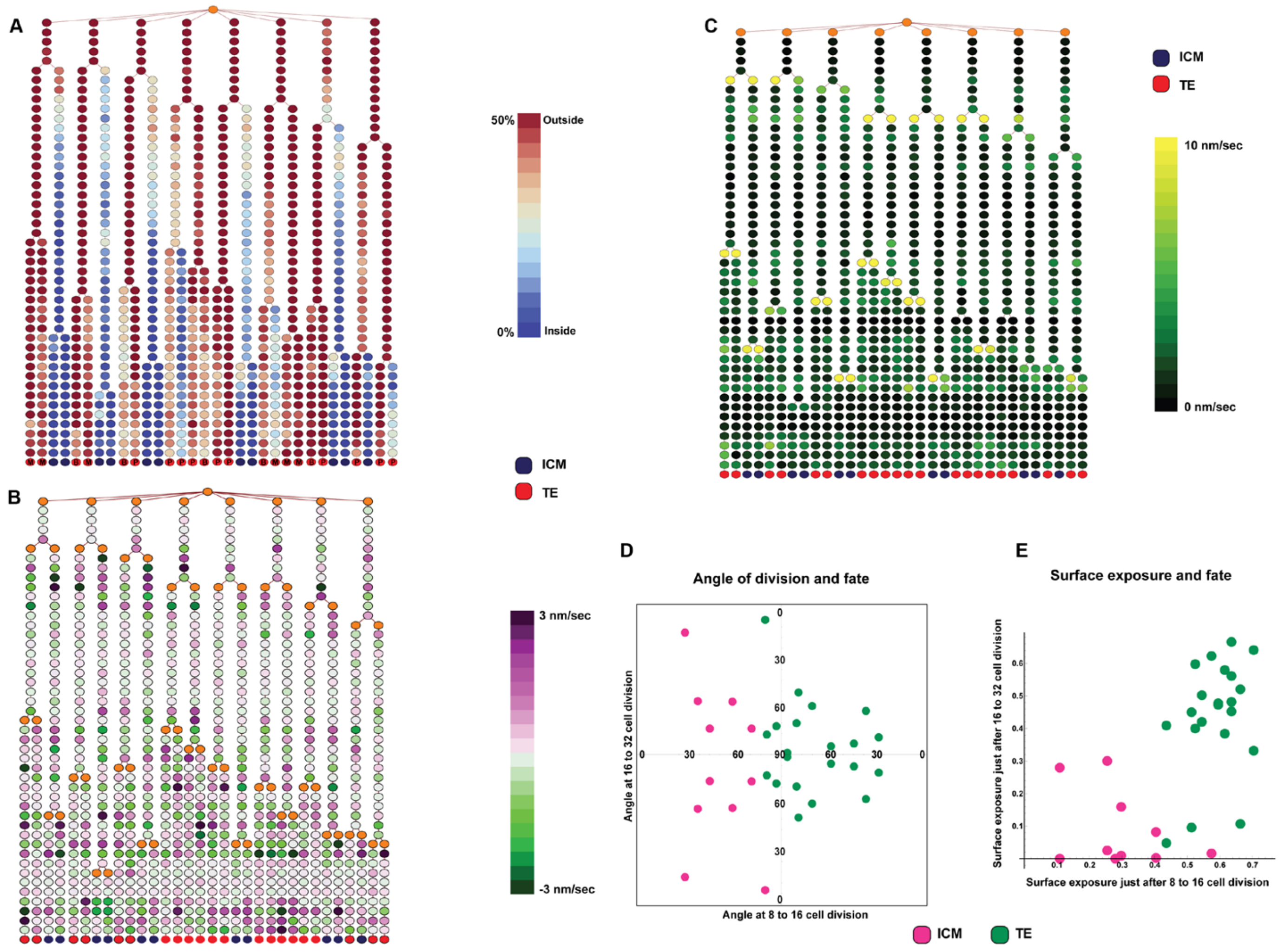

Supplementary Figure S4

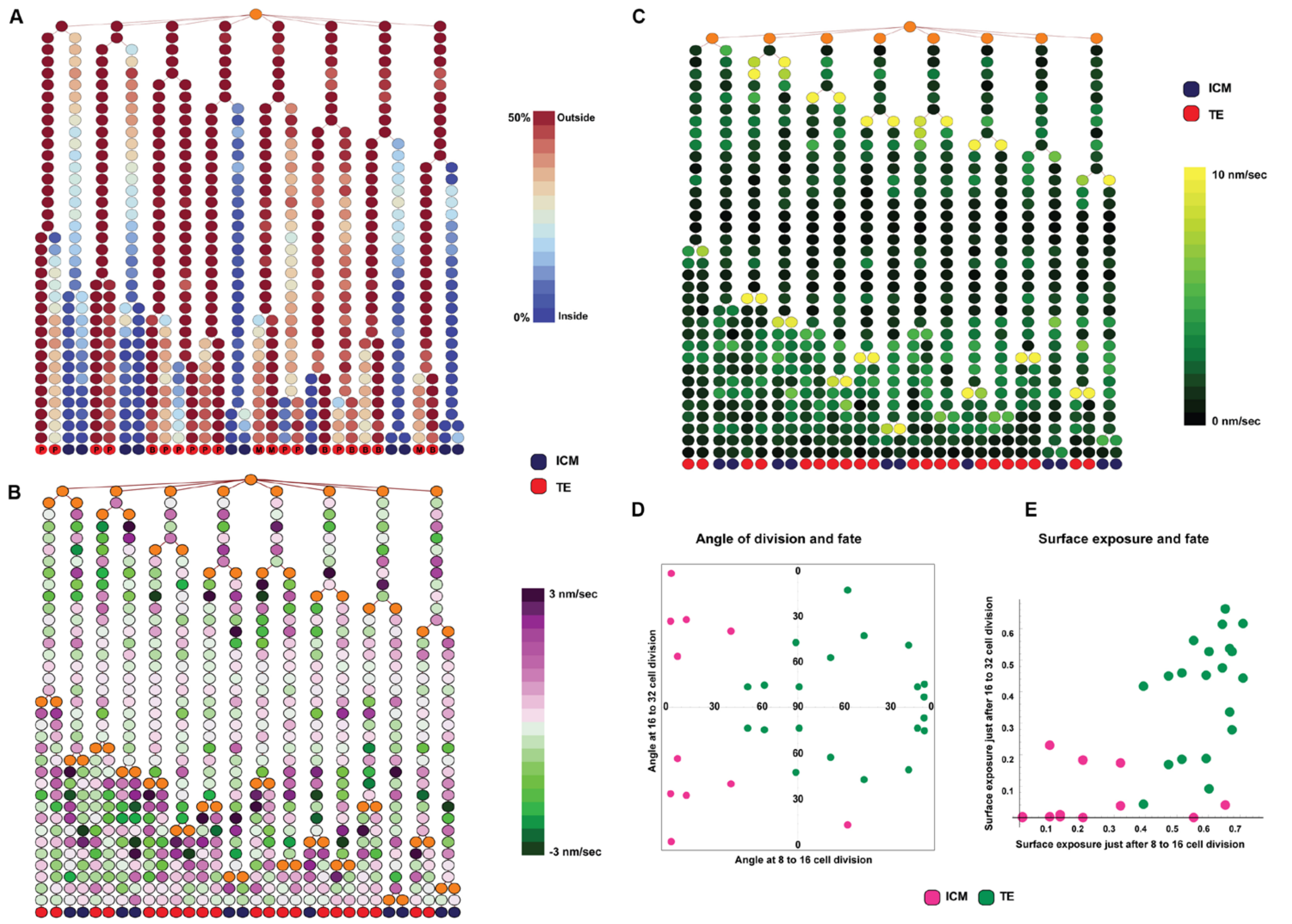

Supplementary Figure S5

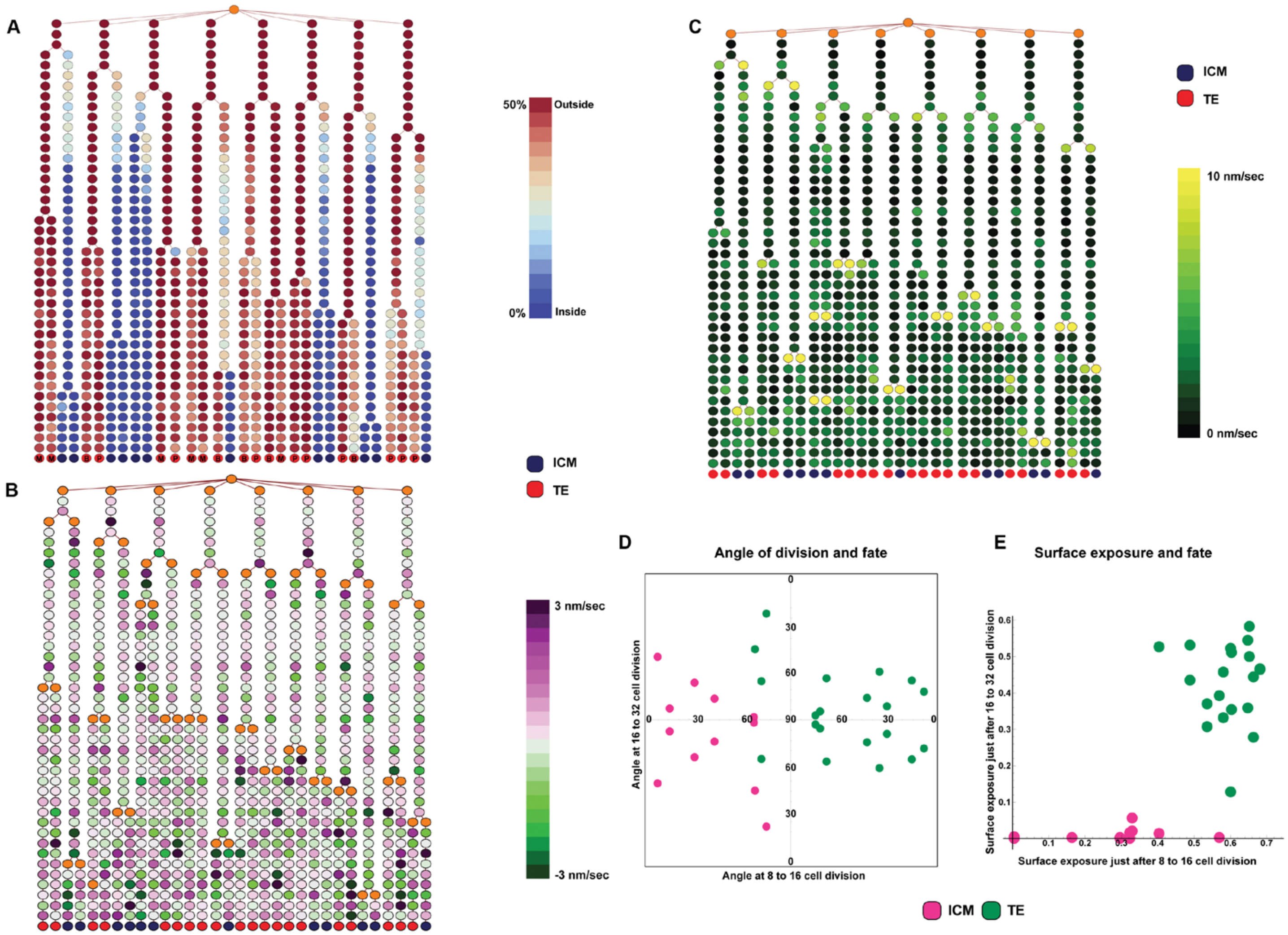

Supplementary Figure S6

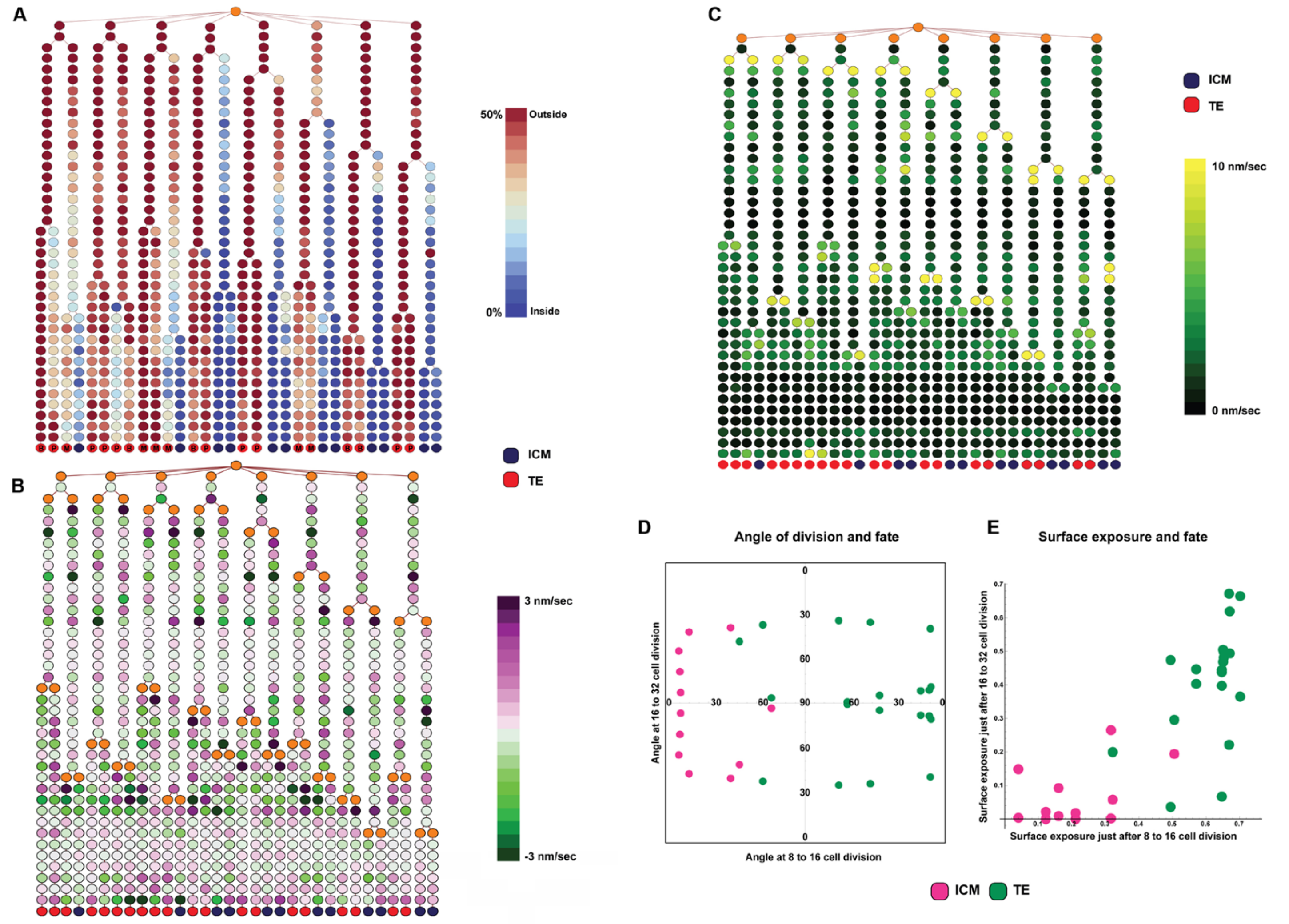

Supplementary Figure S7

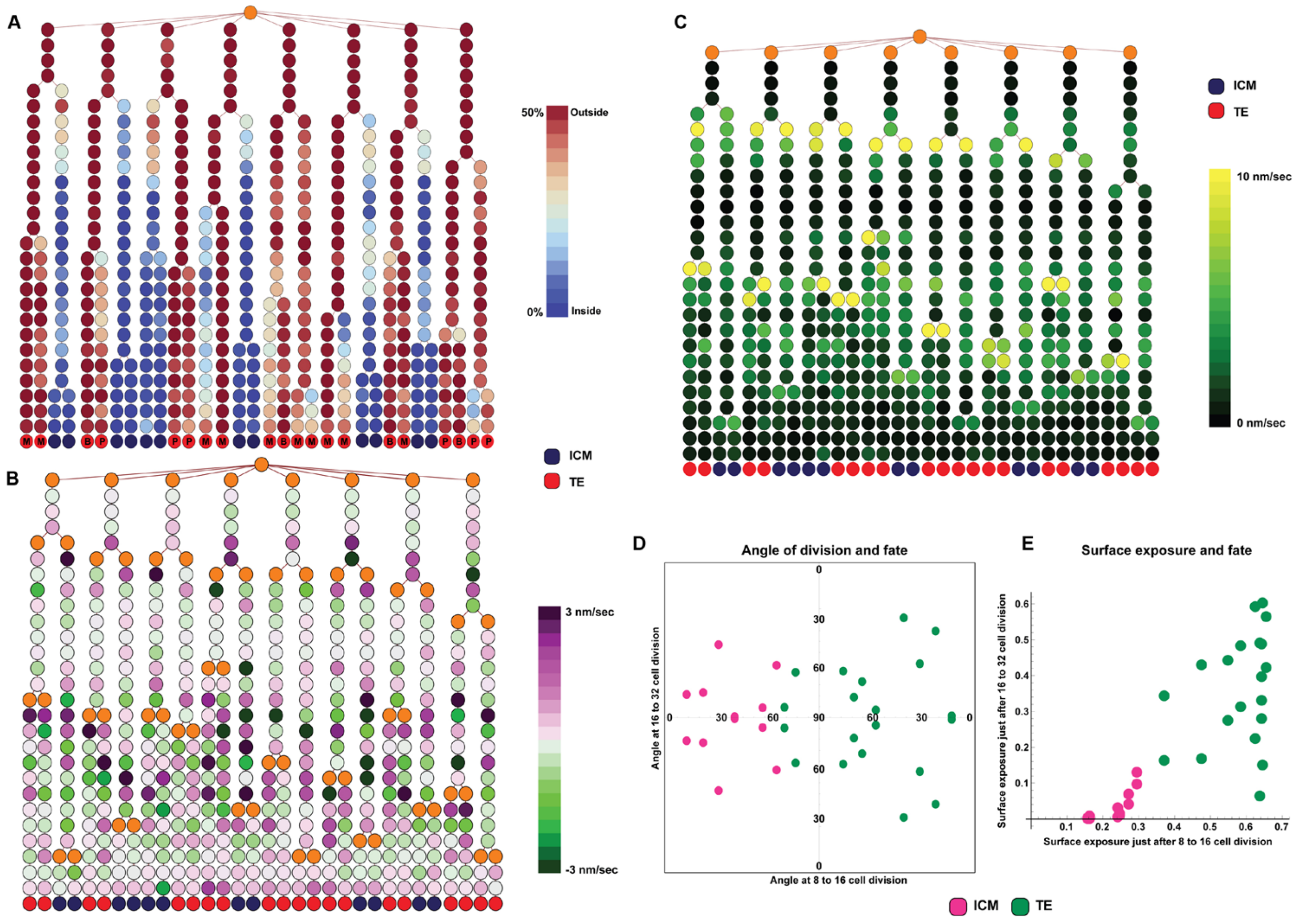

Supplementary Figure S8

Supplementary Figures S3 to S8. Data for each of the individual embryos used in analyses. In all 6 figures, panel A is a lineage tree showing surface exposure, B) shows radial movement, with positive values being outward and negative values being inwards, C) shows overall relative movement, D) plots history of division angle against fate and E) plots history of surface exposure and fate. In panel A, TE cells are labeled ‘M’ for mural trophectoderm, ‘P’ for polar trophectoderm and ‘B’ if they are between the mural and polar trophectoderm.

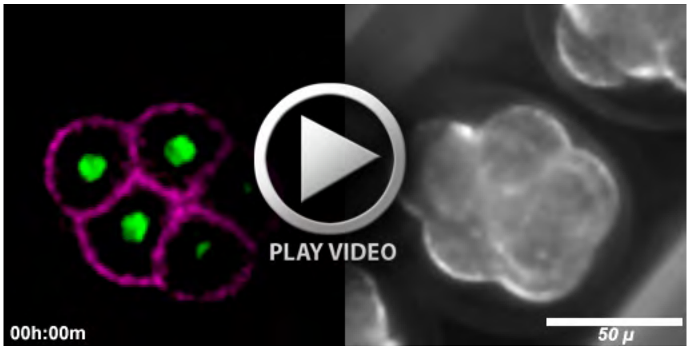

Supplementary Movie 1. Time-lapse sequence of an embryo developing from morula to early blastocyst. The panel at left shows fluorescence and the panel at right shows the bright-field image. The embryo expresses myr-TdTomato in the plasma membrane (magenta) and H2B-GFP in the nucleus (green).

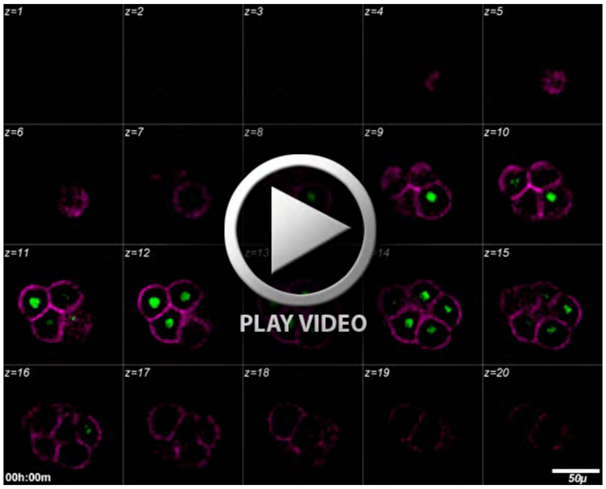

Supplementary Movie 2. Time-lapse sequence of an embryo developing from morula to early blastocyst. Montage of all the z-levels of the embryo shown in movie 1. The embryo expresses myr-TdTomato in the plasma membrane (magenta) and H2B-GFP in the nucleus (green).

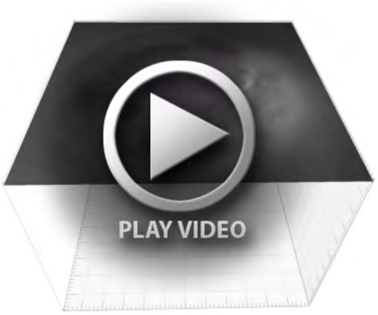

Supplementary Movie 3. Segmentation of embryo blastomeres. Animation illustrating how bitmap image data is converted to vector representations of individual blastomeres. One starts with a 3D (or in our case, 4D) data-set, and outlines blastomeres one at a time across the various focal planes, to segment them. This is repeated across the different time-points, to segment entire lineages.

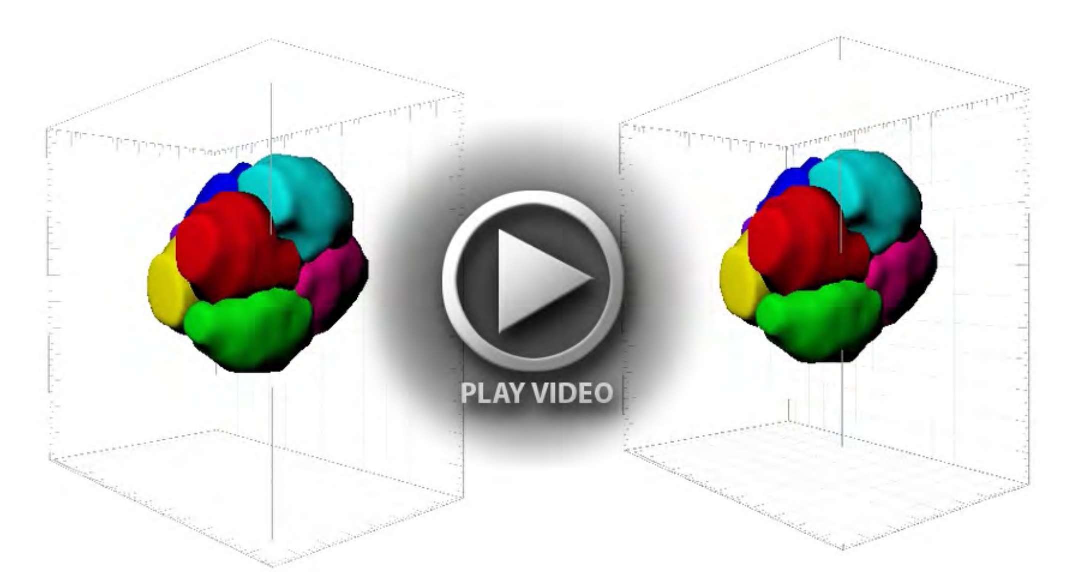

Supplementary Movie 4. Formation of the blastocyst in a ‘digital’ embryo. Representative digital embryo, developing from 8-cell morula to blastocyst stage. Each of the blastomeres at the 8-cell stage is given a unique colour. Upon division, daughters inherit the colour of the mother. The panels at left and right show the same embryo from the same viewpoint, but in the panel at right, all but one blastomere is made translucent, so one can focus on the development and contributions of the single magenta blastomere.

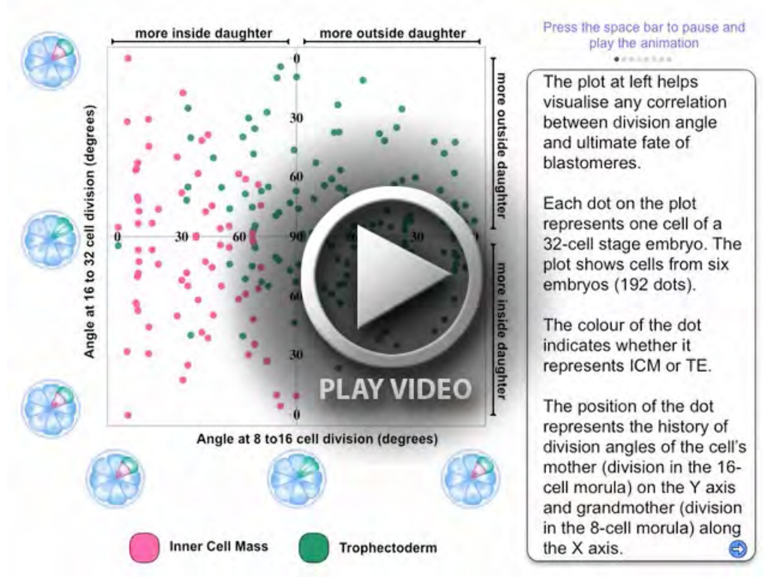

Supplementary Movie 5. Animation describing how the graph in Figure 5 was plotted.

Table S1. Development of imaged embryos transferred into pseudopregnent recipients

Transfer number	Number of embryos transferred		Number of pups weaned	
1	9	3 fluorescent + 6 non-fluorescent	5 (56%)	2 fluorescent + 3 non-fluorescent
2	5	2 fluorescent + 3 non-fluorescent	4 (80%)	2 fluorescent + 2 non-fluorescent
3	10	8 fluorescent + 2 non-fluorescent	8 (80%)	6 fluorescent + 2 non-fluorescent
4 (control)	8	not imaged but cultured in the microscope	6 (75%)	
